# Smoking affects gene expression in blood of patients with ischemic stroke

**DOI:** 10.1002/acn3.50876

**Published:** 2019-08-22

**Authors:** Xiyuan Cheng, Eva Ferino, Heather Hull, Glen C. Jickling, Bradley P. Ander, Boryana Stamova, Frank R. Sharp

**Affiliations:** ^1^ Department of Neurology University of California at Davis Sacramento California; ^2^ Toxicology and Pharmacology Graduate Program University of California at Davis Davis California; ^3^ Department of Neurology University of Alberta Edmonton California

## Abstract

**Objective:**

Though cigarette smoking (CS) is a well‐known risk factor for ischemic stroke (IS), there is no data on how CS affects the blood transcriptome in IS patients.

**Methods:**

We recruited IS‐current smokers (IS‐SM), IS‐never smokers (IS‐NSM), control‐smokers (C‐SM), and control‐never smokers (C‐NSM). mRNA expression was assessed on HTA‐2.0 microarrays and unique as well as commonly expressed genes identified for IS‐SM versus IS‐NSM and C‐SM versus C‐NSM.

**Results:**

One hundred and fifty‐eight genes were differentially expressed in IS‐SM versus IS‐NSM; 100 genes were differentially expressed in C‐SM versus C‐NSM; and 10 genes were common to both IS‐SM and C‐SM (*P* < 0.01; |fold change| ≥ 1.2). Functional pathway analysis showed the 158 IS‐SM‐regulated genes were associated with T‐cell receptor, cytokine–cytokine receptor, chemokine, adipocytokine, tight junction, Jak‐STAT, ubiquitin‐mediated proteolysis, and adherens junction signaling. IS‐SM showed more altered genes and functional networks than C‐SM.

**Interpretation:**

We propose some of the 10 genes that are elevated in both IS‐SM and C‐SM (GRP15, LRRN3, CLDND1, ICOS, GCNT4, VPS13A, DAP3, SNORA54, HIST1H1D, and SCARNA6) might contribute to increased risk of stroke in current smokers, and some genes expressed by blood leukocytes and platelets after stroke in smokers might contribute to worse stroke outcomes that occur in smokers.

## Introduction

Cigarette smoking (CS) is a significant, modifiable risk factor for ischemic stroke (IS).^1^, ^2^, ^3^, ^4^, ^5^, ^6^, ^7^, ^8^, ^9^, ^10^ CS accounts for roughly 15% of all stroke death and has a dose‐response relationship in older age subjects.^11^ Smoking cessation not only rapidly reduces the risk of primary stroke, with the risk almost disappearing within 4 years of smoking cessation, but also improves outcomes of recurrent IS.^4^, ^7^, ^10^, ^11^

Smoking has a variety of detrimental effects on the cerebrovascular and cardiovascular systems by promoting endothelial dysfunction, systemic inflammation, high levels of low‐density lipoprotein cholesterol, atherosclerosis, platelet aggregation, and clot formation.^9^ Thus, smoking increases the rates of stroke as well as myocardial infarction. Herein, we hypothesize that CS induces gene expression changes, especially in inflammation‐related and platelet aggregation‐related genes, resulting in an increased risk of IS.

To address this, we performed a whole blood transcriptome analysis to identify differences and similarities between IS‐SM versus IS‐NSM and C‐SM versus C‐NSM. Some of the genes unique to IS‐SM may explain why smokers tend to have poorer outcomes following IS, and some of the genes common to IS‐SM and C‐SM may contribute to the risk of stroke among smokers. To our knowledge, this study is the first to describe changes of gene expression in whole blood at the whole genome level following ischemic stroke in humans in smokers compared to never smokers.

## Methods and materials

### Study participants

Subjects (*n* = 219) were recruited from the Universities of California at Davis and San Francisco, and the University of Alberta, Canada. These included 42 IS‐current smokers (IS‐SM), 68 IS‐never smokers (IS‐NSM), 23 control‐smokers (C‐SM), and 86 control‐never smokers (C‐NSM). Ethics approval was obtained from the institutional review boards, and written informed consent was obtained from each participant. All procedures followed institutional guidelines. Diagnosis of IS was made by two board‐certified neurologists based on medical history, and computed tomographic (CT) and/or magnetic resonance imaging (MRI) brain scans. Controls include healthy subjects and vascular risk factor subjects. Vascular risk factor subjects had hypertension, diabetes, and/or hypercholesterolemia. Exclusion criteria included prior strokes, active cancer, infection just before or after strokes, or a rheumatological disorder.

Smoking status was determined by questionnaire. Current smokers were those who smoke on average at least one cigarette per day during the past 12 months. Never smokers (both stroke S‐NS and controls C‐NS) had never smoked by self‐report.

### Sample processing and total RNA isolation

One venous blood sample was collected from each IS patient. The time of sample collection ranged from 4.4 to 83.2 h after IS. Blood was drawn into PAX gene tubes (Qiagen) and stored frozen at −80°C until processed.^12^, ^13^

Total RNA was extracted according to protocol (PAXgene blood RNA kit; Qiagen). RNA quality and concentration were measured using Agilent 2100 Bioanalyzer and Nano‐drop, respectively. *A*_260_/*A*_280_ absorbance ratios ≥ 1.8, a 28S/18S rRNA ratio ≥ 1.8, and an RNA integrity number (RIN) ≥8 were used for determining RNA quality. Reverse transcription, amplification, and sample labeling were carried out using Nugen’s Ovation Whole Blood Solution (Nugen Technologies, San Carlos, CA) to generate cDNA for analysis on Affymetrix GeneChip^®^ arrays.

### HTA 2.0 microarray

The Affymetrix HTA 2.0 Gene Chip microarrays (Affymetrix, Santa Clara, CA) were used to measure expression of mRNAs and noncoding RNA. Amplified cDNAs were hybridized to Affymetrix HTA 2.0 Gene Chip microarrays, washed on a Fluidics Station 450 and scanned on a GeneChip Scanner 3000. Samples were randomly assigned to microarray batches to reduce batch effect. Microarray raw gene expression data were saved in CEL files.

### Statistical analysis

Raw gene expression data were input into Partek Flow software (Partek Inc., St. Louis, MO) and normalization performed using robust multichip averaging (RMA). Statistical analyses of mRNA data were performed for (1) IS‐SM versus IS‐NSM and (2) C‐SM versus C‐NSM. Univariate analyses were performed to determine confounding factors, including gender, age, race, and vascular risk factors that might differ between IS‐SM and IS‐NSM, as well as C‐SM and C‐NSM. Fisher’s exact test was used for categorical variables and unpaired *t*‐test for continuous variables. A mixed effect regression model was utilized for differential gene expression analysis, including diagnosis, smoking status, significant factors from the univariate analysis, technical variation (batch), gender and interaction between diagnosis and smoking status. The criteria for determining significantly differentially expressed genes were *P* < 0.01 and |fold change| ≥ 1.2. A fold change of 1.2 was used as in our previous studies to help ensure biologically significant changes and to provide enough genes for functional pathway analysis.^14^, ^15^, ^16^, ^17^

### Functional pathway analyses and cross‐validation analysis

Exploratory Gene Association Networks (EGAN) software was used to analyze the functional networks. Gene networks and pathways were generated according to the Kyoto Encyclopedia of Genes and Genomes (KEGG) database.

Cross‐validation analysis was performed to determine prediction accuracy of the optimal model using forward selection and the k‐nearest neighbor algorithm in Partek Genomics Suite 6.4.^18^ Sensitivity and specificity of the best classifier were calculated.

## Results

### Demographic and clinical characteristics

Demographic and clinical characteristics of the 219 subjects are summarized in Table 1. Average age of IS subjects (years ± SD) was 60.9 ± 12.6 and 75.5% were male. There were 55.5% white, 16.4% black, 7.3% Hispanic, 10 % Asian, and 10.9% other race. Hypertension was present in 78.2%, diabetes in 41.8%, and hypercholesterolemia in 45.5% of IS subjects. For controls, the average age was 56.7 ± 13.2 and 31.2% were male. For controls 56% were white, 11% black, 16.5% Hispanic, 8.3% Asian, and 8.3% other race. 46.8% of controls had hypertension, 11% diabetes, and 29.4% hypercholesterolemia. Age, gender, race, hypertension, diabetes, and hypercholesterolemia were not significantly different (*P* > 0.05) in comparisons of IS‐SM versus IS‐NSM (Table 2) and C‐SM versus C‐NSM (Table 3). In addition, the time‐since stroke onset was not significantly different in the IS‐SM versus IS‐NSM groups (Table 2).

**Table 1:** Summary of characteristics of recruited ischemic stroke patients and control subjects.

Characteristics	IS (*n* = 110)	Control (*n* = 109)	*P*‐value
Age years (Mean ± SD)	60.9 ± 12.6	56.7 ± 13.2	0.02
Gender male *n* (%）	83 (75.5%)	34 (31.2%)	0.0001
Race Caucasian *n* (%)	61 (55.5%)	61 (56%)	1
Black	18 (16.4%)	12 (11%)	0.33
Hispanic	8 (7.3%)	18 (16.5%)	0.04
Asian	11 (10%)	9 (8.3%)	0.82
Other race	12 (10.9%)	9 (8.3%)	0.65
Hypertension *n* (%）	86 (78.2%)	51 (46.8%)	0.0001
Diabetes *n* (%）	46 (41.8%)	12 (11%)	0.0001
Hypercholesterolemia *n* (%）	50 (45.5%)	32 (29.4%)	0.0175
Time postevent, hours (Mean ± SD)	43.7 (22.2)	N/A	N/A
Current Smoker *n* (%）	42 (38.2%)	23 (21.1%)	0.0076
Never Smoker *n* (%）	68 (61.8%)	86 (78.9%)	0.0076

**Table 2:** Demographic variables for smokers with ischemic strokes (IS‐SM) compared to never smokers with ischemic strokes (IS‐NSM).

Characteristics	IS‐SM (*n* = 42)	IS‐NSM (*n* = 68)	*P*‐value
Gender male *n* (%）	33 (78.6%)	50 (73.5%)	0.651
Age years (Mean ± SD)	59.6 ± 10.9	61.8 ± 13.5	0.386
Race Caucasian *n* (%)	25 (59.5%)	36 (52.9%)	0.557
Hypertension *n* (%）	34 (81%)	52 (76.5%)	0.641
Diabetes *n* (%）	18 (42.9%)	28 (41.2%)	1
Hypercholesterolemia *n* (%）	17 (40.5%)	33 (48.5%)	0.437
Time postevent, hours (Mean ± SD)	42 ± 23.2	44.8 ± 21.7	0.526

*P*‐values represent the comparisons of IS‐SM versus IS‐NSM using *t*‐test, Chi‐square, or Fisher’s exact test where appropriate.

**Table 3:** Demographic variables for control‐smokers (C‐SM) compared to control‐never smokers (C‐NSM).

Characteristics	C‐SM (*n* = 23)	C‐NSM (*n* = 86)	*P*‐value
Gender male *n* (%）	10 (43.5%)	24 (27.9%)	0.205
Age years (Mean ± SD)	58.7 ± 9	56.2 ± 14.1	0.423
Race Caucasian *n* (%)	14 (60.9%)	47 (54.7%)	0.643
Hypertension *n* (%）	7 (30.4%)	44 (51.2%)	0.101
Diabetes *n* (%）	3 (13%)	9 (10.5%)	0.714
Hypercholesterolemia *n* (%）	3 (13%)	29 (33.7%)	0.071
Time postevent, hours (Mean ± SD)	N/A	N/A	N/A

*P*‐values represent the comparisons of C‐SM versus C‐NSM using *t*‐test, Chi‐square, or Fisher’s exact test where appropriate.

### Differentially expressed mRNAs, miRNAs, and lncRNAs

**Figure 1:**
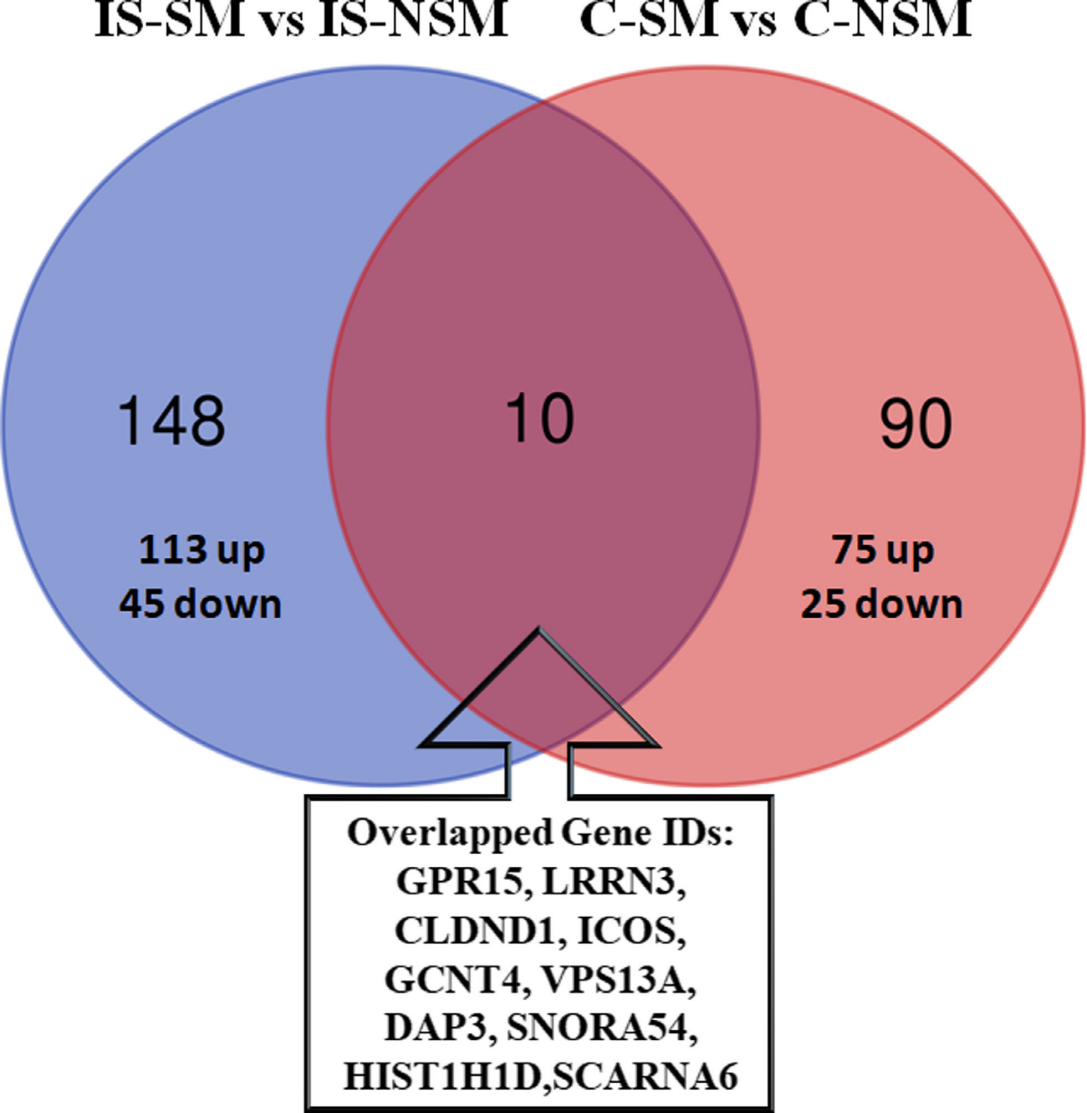
Differentially expressed genes (*P*‐value < 0.01; |fold change| ≥ 1.2) in ischemic stroke‐smokers (IS‐SM) versus ischemic stroke‐never smokers (IS‐NSM), and in control‐smokers (C‐SM) versus control‐never smokers (C‐NSM). Abbreviations: GPR15, G Protein‐Coupled Receptor 15; LRRN3, Leucine Rich Repeat Neuronal 3; CLDND1, Claudin Domain Containing 1; ICOS, Inducible T‐Cell Costimulator; GCNT4, Glucosaminyl (N‐Acetyl) Transferase 4; VPS13A, Vacuolar Protein Sorting 13 Homolog A; DAP3, Death‐Associated Protein 3; SNORA54, Small Nucleolar RNA, H/ACA Box 54; HIST1H1D, Histone Cluster 1 H1 Family Member D; SCARNA6, Small Cajal Body‐Specific RNA 6.

A total of 158 (113 upregulated and 45 downregulated) genes (RNA) were significantly different between IS‐SM and IS‐NSM (*P* < 0.01, |fold change| ≥ 1.2). Almost a quarter of the 158 IS‐SM versus IS‐NSM genes are noncoding RNA (39 genes), including 3 miRNA, 2 long intergenic RNA (LINC), 2 SCARNA, and 32 small nucleolar RNA (SNORA, SNORD, SNRPN). A total of 100 RNA (75 up and 25 down) were significantly altered between C‐SM and C‐NSM (*P* < 0.01, |fold change| ≥ 1.2). Of these 100 RNAs, 21 were noncoding RNAs. Ten genes, including GPR15, LRRN3, CLDND1, ICOS, GCNT4, VPS13A, DAP3, HIST1H1D, SNORA54*, and SCARNA6*, were overexpressed in both the IS‐SM versus IS‐NSM and in C‐IS versus C‐NSM (Fig. 1) (*small noncoding RNAs).

### Functional analysis of regulated mRNAs for IS‐SM versus IS‐NSM and C‐SM versus C‐NSM

**Figure 2:**
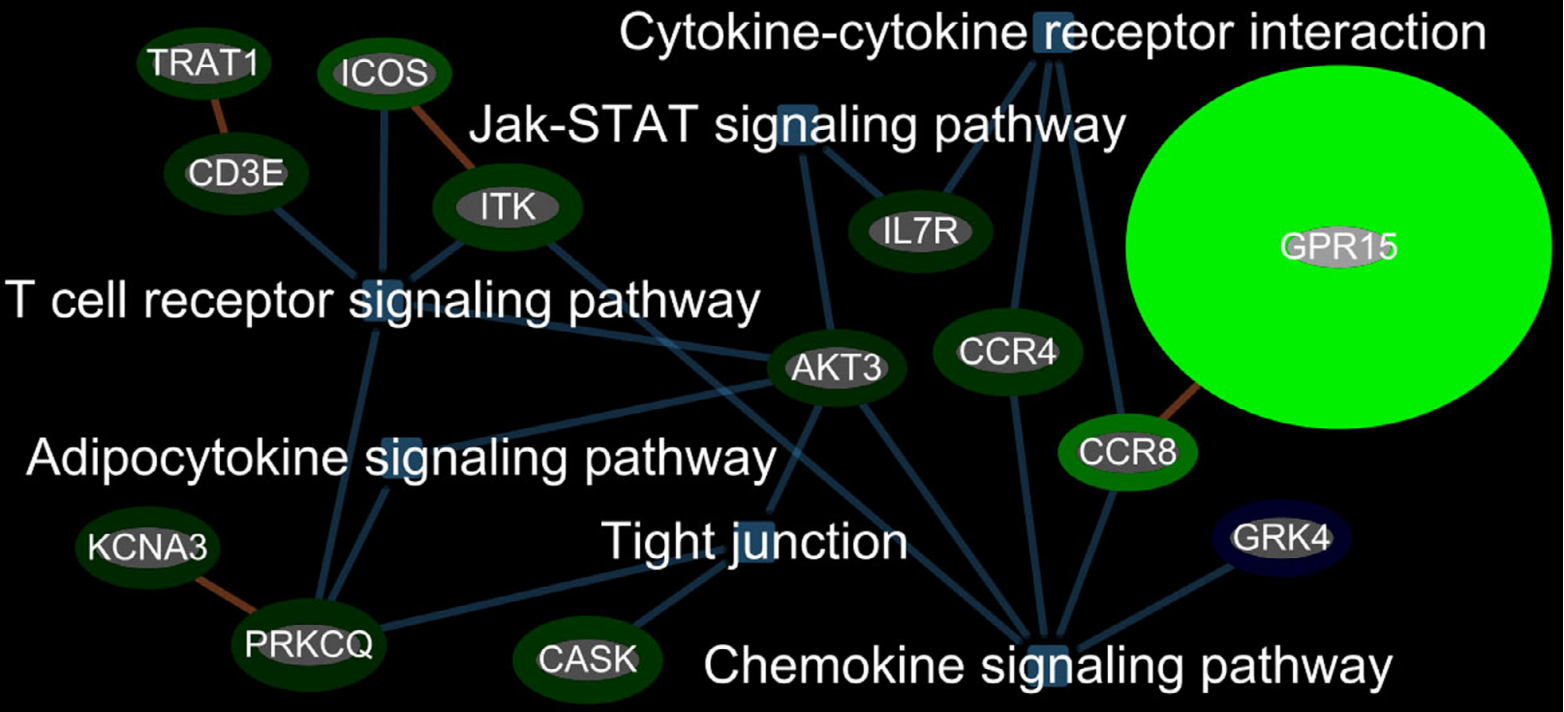
Top inflammatory response and immune response‐related functional pathways for regulated genes associated with IS‐SM when compared to IS‐NSM. Abbreviations: TRAT1, T‐Cell Receptor Associated Transmembrane Adaptor 1; ICOS, Inducible T‐Cell Costimulator; CD3E, CD3e Molecule; ITK, IL2 Inducible T‐Cell Kinase; KCNA3, Potassium Voltage‐Gated Channel Subfamily A Member 3; PRKCQ, Protein Kinase C Theta; CASK, Calcium/Calmodulin‐Dependent Serine Protein Kinase; AKT3, AKT Serine/Threonine Kinase 3; IL7R, Interleukin 7 Receptor; CCR4, C‐C Motif Chemokine Receptor 4; CCR8, C‐C Motif Chemokine Receptor 8; GRK4, G Protein‐Coupled Receptor Kinase 4.

**Figure 3:**
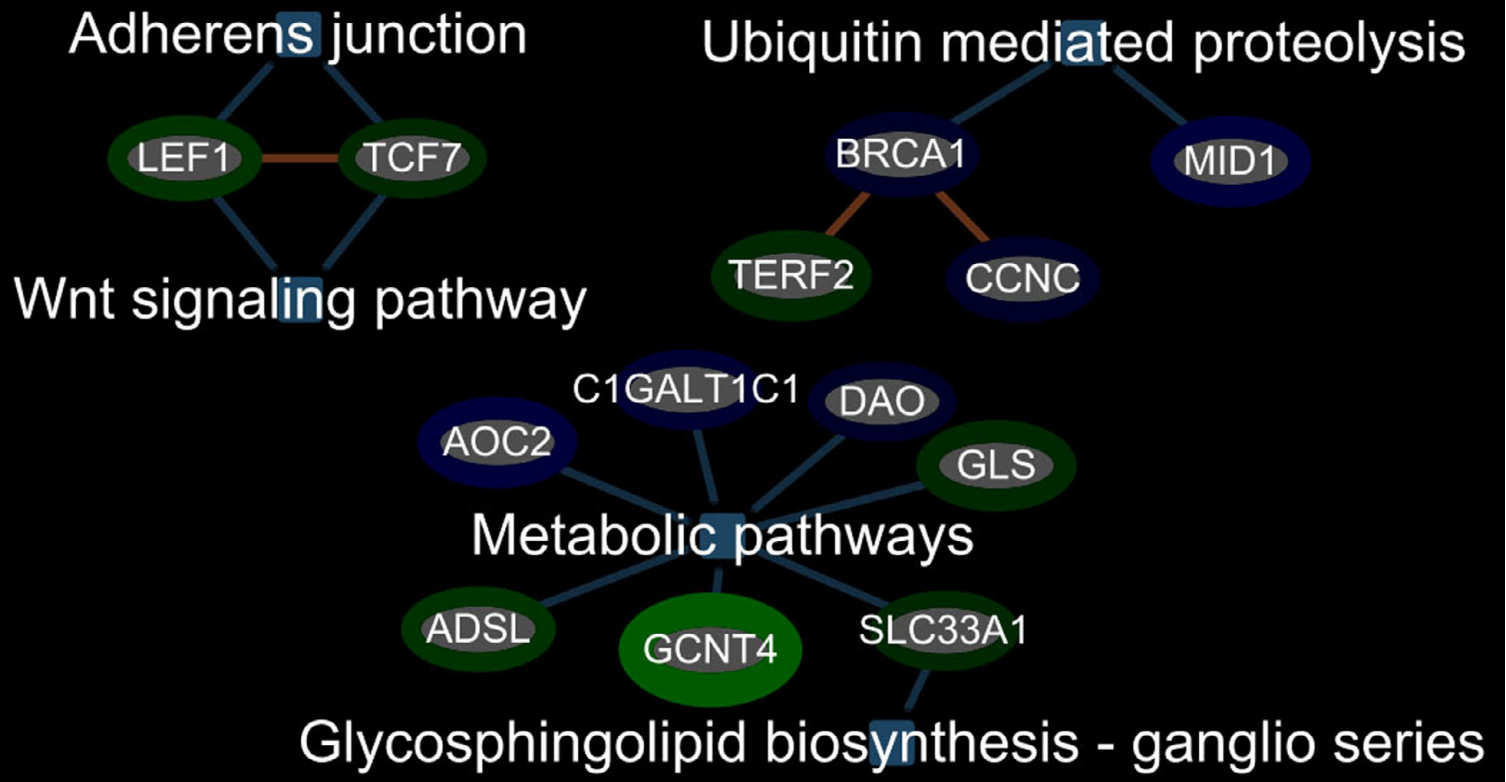
Significant metabolic pathways, Ubiquitin‐mediated proteolysis pathway and Adherens junction and Wnt signaling pathway for regulated genes associated with IS‐SM when compared to IS‐NSM. Abbreviations: LEF1, Lymphoid Enhancer‐Binding Factor 1; TCF7, Transcription Factor 7; BRCA1, BRCA1, DNA Repair Associated; TERF2, Telomeric Repeat‐Binding Factor 2; CCNC, Cyclin C; MID1, Midline 1; AOC2, Amine Oxidase, Copper Containing 2; C1GALT1C1, C1GALT1 Specific Chaperone 1; DAO, D‐Amino Acid Oxidase; GLS, Glutaminase; SLC33A1, Solute Carrier Family 33 Member 1; GCNT4, Glucosaminyl (N‐Acetyl) Transferase 4; ADSL, Adenylosuccinate Lyase.

Functional pathway analysis showed that the 158 genes (113 up and 45 down) differentially expressed in blood of IS‐SM versus IS‐NSM were over‐represented in T‐cell receptor, cytokine–cytokine receptor, chemokine, adipocytokine, tight junction, Jak‐STAT, ubiquitin‐mediated proteolysis, and adherens junction signaling (Figs. 2 and 3). The 100 differentially expressed genes (75 up and 25 down) in blood of C‐SM versus C‐NSM were associated with cell adhesion, antigen processing and presentation, T‐cell receptor, natural killer cell‐medicated cytotoxicity, cytokine–cytokine receptor interaction, apoptosis, and endocytosis signaling pathways (Fig. S1).

The mRNA predictive model that was able to best discriminate IS‐SM from IS‐NSM was generated using a *k*‐nearest neighbor algorithm (*k* = 21) with a normalized correct rate of 79.9% using 27 genes. The sensitivity was 64.3% and specificity was 95.6% for IS‐SM.

## Discussion

In this study, we identified 158 differentially expressed genes associated with IS‐SM compared to IS‐NSM, 100 altered genes associated with C‐SM compared to C‐NSM, and 10 common genes for IS‐SM versus IS‐NSM and C‐SM versus C‐NSM. Some of the 10 common genes (GPR15, LRRN3, CLDND1, ICOS, GCNT4, VPS13A, DAP3, HIST1H1D, SNORA54, and SCARNA6) could be associated with stroke risk since they are expressed in smokers before stroke and in smokers after stroke. For example, regulation of ICOS affects outcomes in experimental rodent stroke models by modulating T cells.^19^ Chorea‐acanthocytosis (ChAc), a neurodegenerative disease, results from loss‐of‐function mutations of the chorein‐encoding gene VPS13A.^20^ Perhaps smoking induced changes in levels of VPS13A would affect red blood cells and predispose to clotting/stroke. HIST1H1D and DAP3 are involved in apoptosis, and SNORA54 is associated with Factor X which plays a key role in clotting (GeneCards). GPR15, LRRN3, and CLDND1 are discussed next.

A previous transcriptome meta‐analysis reported the top 25 smoking‐related genes associated with current smokers versus never smokers.^21^ Comparing our 100 smoking genes identified in C‐SM versus C‐NSM with the top 25 smoking‐related genes identified in this previous meta‐analysis (*n* = 25), we found three genes (GPR15, LRRN3, and CLDND1) overlapped, which is highly significant based on a hypergeometric probability test (*P* < 0.0002). These three genes were also among the 10 common genes differentially expressed in IS‐SM, as well as in C‐SM (Fig. 1, overlap). Notably, smoking is the only condition known to increase the numbers of GPR15+ T cells in blood^22^ which are pro‐inflammatory Th17‐like.^23^ GPR15 is an orphan receptor that is involved in the regulation of the innate immunity and T‐cell trafficking.^24^ GPR15 and LRRN3 have DNA methylation loci in their promoter regions that are reported to be hypomethylated among smokers which might indicate smoking‐induced epigenetic changes.^21^ LRRN3 is an inflammatory regulatory gene related to T‐cell function and immunosenescence whose expression declines with age^25^ and smoking, perhaps resulting in a dysregulated immune system prior to and after stroke. Claudin domain‐containing 1 (CLDND1), also known as claudin‐25, plays a role in the development of cerebrovascular disease in stroke‐prone spontaneously hypertensive rats.^26^ Perhaps smoking induction of CLDND1 is a risk factor for stroke in humans as well.

Some of the 158 genes expressed after stroke in smokers could be associated with the worse stroke outcomes in smokers. It is notable that most of the genes associated with IS‐SM versus IS‐NSM compared to C‐SM versus C‐NSM were different/unique for each. This suggests that smoking has a strong interaction with stroke associated responses in peripheral leukocytes and platelets that is quite different from control‐smokers compared to control‐never smokers.

Of the 158 differentially expressed genes associated with IS‐SM many were in pro‐inflammatory pathways including T‐cell receptor signaling (CD3E, TRAT1, ICOS, ITK, AKT3, PRKCQ, KCNA3), chemokine signaling (GRK4, CCR4, CCR8, AKT3 ITK, GPR15), cytokine–cytokine receptor interaction (IL7R, CCR4, CCR8), adipocytokine signaling (AKT3, PRKCQ, KCNA3), hypertension (GRK4), and tight junction pathways (CASK, AKT3). These pathways which are activated in peripheral blood leukocytes, platelets, and other blood cells could promote more clotting and oxidative stress and contribute to smoking‐induced injury in brain and worse stroke outcomes in smokers.

Many of the IS‐SM genes were associates either with T lymphocytes or T‐cell receptor (TCR) signaling including CD3E, ICOS, PRKCQ, LEF1, TCF7, and IL7R. T cells increase inflammation in atherosclerotic plaques and contribute to lesion development, and T cells infiltrate ischemic brain and may be beneficial or harmful.^27^, ^28^ For example, an anti‐CD3 antibody reduced atherosclerotic plaque development in mice.^29^ Patients with myocardial infarction (MI) and/or stable angina who were diagnosed with atherosclerosis had associated decreases of ICOS+ T cell subset and Treg cells, suggesting a possible role for these cells.^30^ A protective role of ICOS was observed in ApoE‐KO mouse model.^31^ Protein Kinase C Theta (PRKCQ) is a serine/threonine protein kinase expressed in T lymphocytes and modulates proliferation and cytokine production and is required for T‐cell driven inflammatory responses.^32^, ^33^ PRKCQ is also highly expressed in platelets and positively regulates thrombin‐mediated platelet activation and aggregation.^34^ Interleukin receptor IL7R was upregulated in the peripheral blood of IS‐SM. IL7 regulates T‐cell differentiation, survival, and homeostasis, which mediates inflammation upon binding to IL7R.^35^ Increased gene expression of IL7R and lnc‐IL7R are observed in many inflammatory conditions including atherosclerotic plaques in humans.^36^, ^37^, ^38^, ^39^ Thus, the dysregulation of some of the above T‐cell receptor signaling pathways could contribute to worsened ischemic brain injury in smokers.

CCR4 and CCR8 are also upregulated in IS‐SM. These are CC chemokine receptors that mediate leukocytes chemotaxis and play regulatory roles in the inflammatory response upon interaction with their chemokines. Atherosclerosis is associated with the recruitment of monocytes and T lymphocytes to the vascular wall of blood vessels. The dendritic cell‐derived chemokine CCL17 is a ligand for CCR4. An atherosclerosis‐prone mice study showed that CCL17 deficiency resulted in a reduction of atherosclerosis, which was dependent on Tregs.^40^, ^41^ Another study found that CCR4 expression was decreased by simvastatin accompanied by anti‐inflammation and lipid‐lowering effects in human endothelial cells and macrophages.^42^ These findings suggested CCR4 is involved in promoting atherosclerosis. In addition to the role in atherosclerosis, studies have shown that CCR4 was expressed on platelets and triggered platelet activation and aggregation via binding to its ligands including macrophage‐derived chemokine in Th2 diseases such as asthma and atopic dermatitis.^43^, ^44^

CCR8 is found on monocytes, T lymphocytes, endothelial cells, and vascular smooth muscle cells in human.^45^, ^46^, ^47^ These cells produce CCR8 ligand, CCL1/I‐309, mediating the effects of Lipoprotein(a) in atherosclerosis. CCR8 was also identified on endothelium of human atherosclerotic plaques and induced angiogenesis when interacting with CCL1.^45^, ^48^ These data showed that CCR8, an endothelial receptor, may regulate endothelial function and participate in arterial vessel wall pathology. In the current study, both CCR4 and CCR8 are upregulated in IS current smoker patients, which may suggest a role of current smoking in increasing stroke injury by promoting atherosclerosis and platelet aggregation.

Smoking also activates the Wnt signaling pathway via LEF1 and TCF7 which modulate adherens junctions. LEF1 is a transcriptional repressor which activates HDACs which downregulate expression of many genes important in the pathogenesis of stroke.^49^ Indeed, HDAC inhibitors in humans can decrease the risk of stroke after a previous stroke or TIA.^50^ Thus, smoking activation of LEF1 would be expected to activate HDACs which could worsen stroke outcomes.

There were many noncoding RNAs (ncRNA) that were associated with stroke in smokers (IS‐SM). Many of the ncRNA, including microRNA and long noncoding RNA, can regulate mRNA expression. As an example, SNORA54 appears to modify expression of Factor X and thus could play a role in clotting related to smoking‐associated strokes (GeneCards). Another example is SCARNA6, a small Cajal body‐specific RNA which are in a class of small nucleolar RNAs (snoRNAs) that localize to the Cajal body, a nuclear organelle involved in the biogenesis of small nuclear ribonucleoproteins (snRNPs or snurps). ScaRNAs guide methylation and pseudouridylation of RNA polymerase II transcribed spliceosomal RNAs U1, U2, U4, U5, and U12 to modulate splicing which might be another impact of smoking in stroke.^51^

One limitation is small sample size. Future larger studies will be required to validate the findings in a separate cohort. Another limitation is the inability to prove causal relationship between smoking, gene expression and clinical outcomes in IS patients due to this being cross‐sectional study. While the gene profiling provides evidence for hypothesis generation, in vitro and in vivo functional experiments need to be performed to establish causality that the identified genes represent either stroke risk genes or represent genes that contribute to smoking‐induced worsening of stroke.

## Conclusions

These studies provide a novel list of targets to possibly decrease stroke risk or improve stroke outcomes in smokers.

## Conflict of Interest

There is no conflict of interest to report for any of the authors.

## Supporting information


**Figure S1.** Top functional pathways for regulated genes in blood associated with C‐SM compared to C‐NSM.

**Table S1.** 158 Differentially expressed genes (*P*‐value < 0.01; |fold change| ≥ 1.2) in ischemic stroke‐smokers (IS‐SM) versus ischemic stroke‐never smokers (IS‐NSM).
**Table S2.** 100 Differentially expressed genes (*P*‐value < 0.01; |fold change| ≥ 1.2) in control‐smokers (C‐SM) versus control‐never smokers (C‐NSM).
**Table S3.** Top functional pathways for regulated genes associated with IS‐SM when compared to IS‐NSM.
**Table S4.** Top functional pathways for regulated genes associated with C‐SM compared to C‐NSM.

## References

[1] Benjamin EJ , Blaha MJ , Chiuve SE , et al. Heart disease and stroke statistics‐2017 update: a report from the American Heart Association. Circulation 2017;135:e146–e603. 28122885 10.1161/CIR.0000000000000485 PMC5408160

[2] Boehme A . Smoking cessation and secondary stroke prevention. Neurology 2017;89:1656–1657. 28887376 10.1212/WNL.0000000000004530

[3] Ding D . Deleterious effect of smoking on ischemic stroke outcomes: implications for the role of chronic inflammation on atherosclerotic plaque pathogenesis. J Stroke Cerebrovasc Dis 2014;23:596–597. 24630131 10.1016/j.jstrokecerebrovasdis.2013.12.034

[4] Fagerstrom K . The epidemiology of smoking: health consequences and benefits of cessation. Drugs 2002;62(Suppl 2):1–9. 10.2165/00003495-200262002-00001 12109931

[5] Kumagai N , Okuhara Y , Iiyama T , et al. Effects of smoking on outcomes after acute atherothrombotic stroke in Japanese men. J Neurol Sci 2013;335:164–168. 24112970 10.1016/j.jns.2013.09.023

[6] Nordahl H , Osler M , Frederiksen BL , et al. Combined effects of socioeconomic position, smoking, and hypertension on risk of ischemic and hemorrhagic stroke. Stroke 2014;45:2582–2587. 25123220 10.1161/STROKEAHA.114.005252

[7] Robbins AS , Manson JE , Lee IM , et al. Cigarette smoking and stroke in a cohort of U.S. male physicians. Ann Intern Med 1994;120:458–462. 8311368 10.7326/0003-4819-120-6-199403150-00002

[8] Shah RS , Cole JW . Smoking and stroke: the more you smoke the more you stroke. Expert Rev Cardiovasc Ther 2010;8:917–932. 20602553 10.1586/erc.10.56 PMC2928253

[9] Siasos G , Tsigkou V , Kokkou E , et al. Smoking and atherosclerosis: mechanisms of disease and new therapeutic approaches. Curr Med Chem 2014;21:3936–3948. 25174928 10.2174/092986732134141015161539

[10] Song YM , Cho HJ . Risk of stroke and myocardial infarction after reduction or cessation of cigarette smoking: a cohort study in korean men. Stroke 2008;39:2432–2438. 18617660 10.1161/STROKEAHA.107.512632

[11] Meschia JF , Bushnell C , Boden‐Albala B , et al. Guidelines for the primary prevention of stroke: a statement for healthcare professionals from the American Heart Association/American Stroke Association. Stroke 2014;45:3754–3832. 25355838 10.1161/STR.0000000000000046 PMC5020564

[12] Stamova B , Ander BP , Jickling G , et al. The intracerebral hemorrhage blood transcriptome in humans differs from the ischemic stroke and vascular risk factor control blood transcriptomes. J Cereb Blood Flow Metab 2018;13:271678X18769513. [Epub ahead of print] 10.1177/0271678X18769513 PMC6727143 29651892

[13] Jickling GC , Stamova B , Ander BP , et al. Prediction of cardioembolic, arterial, and lacunar causes of cryptogenic stroke by gene expression and infarct location. Stroke 2012;43:2036–2041. 22627989 10.1161/STROKEAHA.111.648725 PMC3422649

[14] Weinsheimer SM , Xu H , Achrol AS , et al. Gene expression profiling of blood in brain arteriovenous malformation patients. Transl Stroke Res 2011;2:575–587. 22184505 10.1007/s12975-011-0103-3 PMC3241209

[15] Dykstra‐Aiello C , Jickling GC , Ander BP , et al. Altered expression of long noncoding RNAs in blood after ischemic stroke and proximity to putative stroke risk loci. Stroke 2016;47:2896–2903. 27834745 10.1161/STROKEAHA.116.013869 PMC5127755

[16] Stamova B , Tian Y , Jickling G , et al. The X‐chromosome has a different pattern of gene expression in women compared with men with ischemic stroke. Stroke 2012;43:326–334. 22052522 10.1161/STROKEAHA.111.629337 PMC3938328

[17] Shroff N , Ander BP , Zhan X , et al. HDAC9 polymorphism alters blood gene expression in patients with large vessel atherosclerotic stroke. Transl Stroke Res 2019;10:19–25. 29651704 10.1007/s12975-018-0619-x PMC6186202

[18] Stamova B , Xu H , Jickling G , et al. Gene expression profiling of blood for the prediction of ischemic stroke. Stroke 2010;41:2171–2177. 20798371 10.1161/STROKEAHA.110.588335 PMC2987675

[19] Luo Y , Yang Y , Zhang H , et al. Effect of inducible co‐stimulatory molecule siRNA in cerebral infarction rat models. Med Sci Monit 2015;21:3003–3007. 26436531 10.12659/MSM.894477 PMC4599179

[20] Lang F , Pelzl L , Schols L , et al. Neurons, erythrocytes and beyond ‐the diverse functions of chorein. Neurosignals 2017;25:117–126. 29179176 10.1159/000485457

[21] Huan T , Joehanes R , Schurmann C , et al. A whole‐blood transcriptome meta‐analysis identifies gene expression signatures of cigarette smoking. Hum Mol Genet 2016;25:4611–4623. 28158590 10.1093/hmg/ddw288 PMC5975607

[22] Bauer M , Hackermuller J , Schor J , et al. Specific induction of the unique GPR15 expression in heterogeneous blood lymphocytes by tobacco smoking. Biomarkers 2019;24:217–224. 30387691 10.1080/1354750X.2018.1539769

[23] Ammitzboll C , von Essen MR , Bornsen L , et al. GPR15(+) T cells are Th17 like, increased in smokers and associated with multiple sclerosis. J Autoimmun 2019;97:114–121. 30245027 10.1016/j.jaut.2018.09.005

[24] Koks S , Koks G . Activation of GPR15 and its involvement in the biological effects of smoking. Exp Biol Med (Maywood) 2017;242:1207–1212. 28423922 10.1177/1535370217703977 PMC5478000

[25] Cho YE , Latour LL , Kim H , et al. Older age results in differential gene expression after mild traumatic brain injury and is linked to imaging differences at acute follow‐up. Front Aging Neurosci 2016;8:168. 27468266 10.3389/fnagi.2016.00168 PMC4942460

[26] Matsuoka H , Tamura A , Kinehara M , et al. Levels of tight junction protein CLDND1 are regulated by microRNA‐124 in the cerebellum of stroke‐prone spontaneously hypertensive rats. Biochem Biophys Res Commun 2018;498:817–823. 29530526 10.1016/j.bbrc.2018.03.063

[27] Gotsman I , Sharpe AH , Lichtman AH . T‐cell costimulation and coinhibition in atherosclerosis. Circ Res 2008;103:1220–1231. 19028921 10.1161/CIRCRESAHA.108.182428 PMC2662382

[28] Lehmann J , Hartig W , Seidel A , et al. Inflammatory cell recruitment after experimental thromboembolic stroke in rats. Neuroscience 2014;279:139–154. 25168731 10.1016/j.neuroscience.2014.08.023

[29] Steffens S , Burger F , Pelli G , et al. Short‐term treatment with anti‐CD3 antibody reduces the development and progression of atherosclerosis in mice. Circulation 2006;114:1977–1984. 17043169 10.1161/CIRCULATIONAHA.106.627430

[30] Ghourbani Gazar S , Andalib A , Hashemi M , et al. CD4(+)Foxp3(+) Treg and its ICOS(+) subsets in patients with myocardial infarction. Iran J Immunol 2012;9:53–60. 22426168 10.22034/iji.2012.16857

[31] Afek A , Harats D , Roth A , et al. A functional role for inducible costimulator (ICOS) in atherosclerosis. Atherosclerosis 2005;183:57–63. 15941568 10.1016/j.atherosclerosis.2005.03.040

[32] Curnock A , Bolton C , Chiu P , et al. Selective protein kinase Ctheta (PKCtheta) inhibitors for the treatment of autoimmune diseases. Biochem Soc Trans 2014;42:1524–1528. 25399564 10.1042/BST20140167

[33] Nagahama K , Ogawa A , Shirane K , et al. Protein kinase C theta plays a fundamental role in different types of chronic colitis. Gastroenterology 2008;134:459–469. 18155708 10.1053/j.gastro.2007.11.005

[34] Cohen S , Braiman A , Shubinsky G , et al. PKCtheta is required for hemostasis and positive regulation of thrombin‐induced platelet aggregation and alpha‐granule secretion. Biochem Biophys Res Commun 2009;385:22–27. 19433059 10.1016/j.bbrc.2009.05.021

[35] Li R , Paul A , Ko KW , et al. Interleukin‐7 induces recruitment of monocytes/macrophages to endothelium. Eur Heart J 2012;33:3114–3123. 21804111 10.1093/eurheartj/ehr245 PMC3598429

[36] Willis CR , Seamons A , Maxwell J , et al. Interleukin‐7 receptor blockade suppresses adaptive and innate inflammatory responses in experimental colitis. J Inflamm (Lond) 2012;9:39. 23057802 10.1186/1476-9255-9-39 PMC3551718

[37] Moreno‐Viedma V , Amor M , Sarabi A , et al. Common dysregulated pathways in obese adipose tissue and atherosclerosis. Cardiovasc Diabetol 2016;15:120. 27561966 10.1186/s12933-016-0441-2 PMC5000404

[38] Yamazaki M , Yajima T , Tanabe M , et al. Mucosal T cells expressing high levels of IL‐7 receptor are potential targets for treatment of chronic colitis. J Immunol 2003;171:1556–1563. 12874249 10.4049/jimmunol.171.3.1556

[39] Cui H , Xie N , Tan Z , et al. The human long noncoding RNA lnc‐IL7R regulates the inflammatory response. Eur J Immunol 2014;44:2085–2095. 24723426 10.1002/eji.201344126 PMC4107034

[40] Weber C , Meiler S , Doring Y , et al. CCL17‐expressing dendritic cells drive atherosclerosis by restraining regulatory T cell homeostasis in mice. J Clin Invest 2011;121:2898–2910. 21633167 10.1172/JCI44925 PMC3223829

[41] Globisch T , Steiner N , Fulle L , et al. Cytokine‐dependent regulation of dendritic cell differentiation in the splenic microenvironment. Eur J Immunol 2014;44:500–510. 24136200 10.1002/eji.201343820

[42] Veillard NR , Braunersreuther V , Arnaud C , et al. Simvastatin modulates chemokine and chemokine receptor expression by geranylgeranyl isoprenoid pathway in human endothelial cells and macrophages. Atherosclerosis 2006;188:51–58. 16321392 10.1016/j.atherosclerosis.2005.10.015

[43] Abi‐Younes S , Si‐Tahar M , Luster AD . The CC chemokines MDC and TARC induce platelet activation via CCR43. Thromb Res 2001;101:279–289. 11248289 10.1016/s0049-3848(00)00402-3

[44] Clemetson KJ , Clemetson JM , Proudfoot AE , et al. Functional expression of CCR44, CCR44, CCR44, and CXCR44 chemokine receptors on human platelets. Blood 2000;96:4046–4054. 11110672

[45] Haque NS , Zhang X , French DL , et al. CC chemokine I‐309 is the principal monocyte chemoattractant induced by apolipoprotein(a) in human vascular endothelial cells. Circulation 2000;102:786–792. 10942748 10.1161/01.cir.102.7.786

[46] Soler D , Chapman TR , Poisson LR , et al. CCR46 expression identifies CD4 memory T cells enriched for FOXP3+ regulatory and Th2 effector lymphocytes. J Immunol 2006;177:6940–6951. 17082609 10.4049/jimmunol.177.10.6940

[47] Freeman CM , Chiu BC , Stolberg VR , et al. CCR47 is expressed by antigen‐elicited, IL‐10‐producing CD4+CD25+ T cells, which regulate Th2‐mediated granuloma formation in mice. J Immunol 2005;174:1962–1970. 15699124 10.4049/jimmunol.174.4.1962 PMC1599789

[48] Harpel PC , Haque NS . Chemokine receptor‐8: potential role in atherogenesis. Isr Med Assoc J 2002;4:1025–1027. 12489497

[49] Nagathihalli NS , Massion PP , Gonzalez AL , et al. Smoking induces epithelial‐to‐mesenchymal transition in non‐small cell lung cancer through HDAC‐mediated downregulation of E‐cadherin. Mol Cancer Ther 2012;11:2362–2372. 22933707 10.1158/1535-7163.MCT-12-0107 PMC3969334

[50] Brookes RL , Crichton S , Wolfe CDA , et al. Sodium valproate, a histone deacetylase inhibitor, is associated with reduced stroke risk after previous ischemic stroke or transient ischemic attack. Stroke 2018;49:54–61. 29247141 10.1161/STROKEAHA.117.016674 PMC5753817

[51] Cao T , Rajasingh S , Samanta S , et al. Biology and clinical relevance of noncoding sno/scaRNAs. Trends Cardiovasc Med 2018;28:81–90. 28869095 10.1016/j.tcm.2017.08.002 PMC5762389

