# Supplementary figures and images for: Smoking affects gene expression in blood of patients with ischemic stroke
Source: Ann Clin Transl Neurol. 2019 Aug 22;6(9):1748–56. doi: 10.1002/acn3.50876 (PMC6764500; doi:10.1002/acn3.50876)

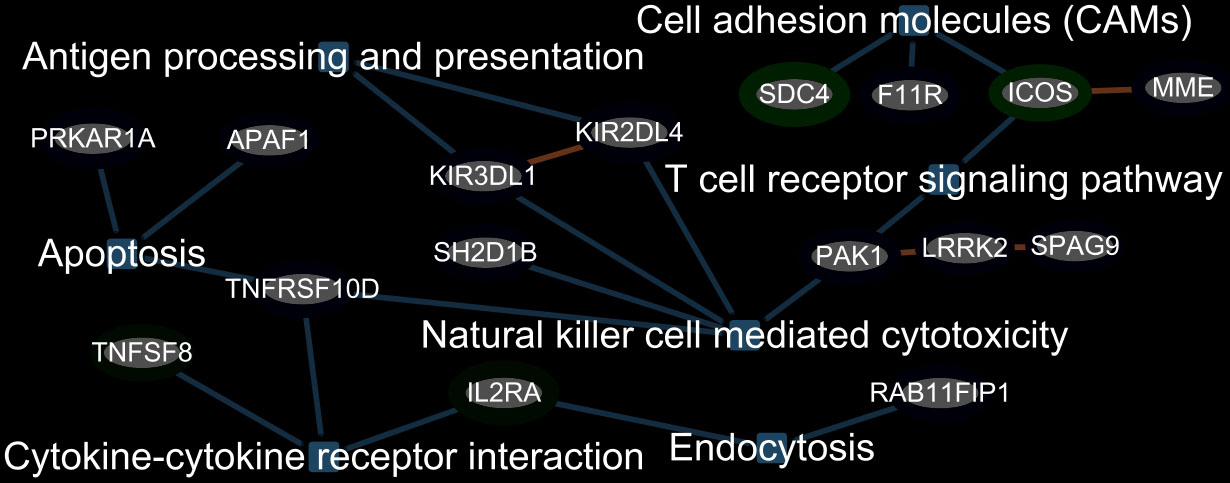

Supplement: Supplementary file 1 — Figure S1. Top functional pathways for regulated genes in blood associated with C‐SM compared to C‐NSM. [file ACN3-6-1748-s001.tif]
