# Supplementary material for: Smoking affects gene expression in blood of patients with ischemic stroke
Source: Ann Clin Transl Neurol. 2019 Aug 22;6(9):1748–56. doi: 10.1002/acn3.50876 (PMC6764500; doi:10.1002/acn3.50876)
Supplement: Supplementary file 2 — Table S1. 158 Differentially expressed genes (P‐value < 0.01; |fold change| ≥ 1.2) in ischemic stroke‐smokers (IS‐SM) versus ischemic stroke‐never smokers (IS‐NSM). Table S2. 100 Differentially expressed genes (P‐value < 0.01; |fold change| ≥ 1.2) in control‐smokers (C‐SM) versus control‐never smokers (C‐NSM). Table S3. Top functional pathways for regulated genes associated with IS‐SM when compared to IS‐NSM. Table S4. Top functional pathways for regulated genes associated with C‐SM compared to C‐NSM. [file ACN3-6-1748-s002.docx]

SUPPLEMENTAL MATERIAL

**Supplemental Table 1.** 158 Differentially expressed genes (p-value <0.01; |fold change| ≥ 1.2) in ischemic stroke-smokers (IS-SM) vs ischemic stroke-never smokers (IS-NSM).

| Gene ID | P-value (IS-SM vs IS-NSM) | Fold change (IS-SM vs IS-NSM) |
| --- | --- | --- |
| GPR15 | 7.38E-27 | 6.83E+00 |
| LRRN3 | 4.56E-08 | 2.10E+00 |
| GCNT4 | 2.81E-05 | 2.73E+00 |
| COL8A2 | 6.11E-05 | 1.80E+00 |
| MIR3687-2 | 6.81E-05 | 1.53E+00 |
| MIR3687-1 | 6.81E-05 | 1.53E+00 |
| IGIP | 1.54E-04 | 1.24E+00 |
| PRKCQ | 1.72E-04 | 1.28E+00 |
| CLDND1 | 1.74E-04 | 1.27E+00 |
| SNRPN | 1.91E-04 | 1.30E+00 |
| LOC100507406 | 2.17E-04 | 1.79E+00 |
| IL32 | 2.30E-04 | 1.54E+00 |
| PWARSN | 3.30E-04 | 2.30E+00 |
| ITK | 3.64E-04 | 1.39E+00 |
| SNORD45B | 4.20E-04 | 2.15E+00 |
| CASK | 4.26E-04 | 1.53E+00 |
| PRKCQ-AS1 | 4.67E-04 | 1.24E+00 |
| SNORD21 | 6.40E-04 | 1.61E+00 |
| CCR4 | 6.75E-04 | 1.56E+00 |
| SLAMF1 | 6.79E-04 | 1.48E+00 |
| TSHZ2 | 7.67E-04 | 2.25E+00 |
| TEX13A | 8.20E-04 | 1.26E+00 |
| SNORA5B | 8.21E-04 | 1.75E+00 |
| CBX3P2 | 8.38E-04 | -1.61E+00 |
| SNORD50A | 8.87E-04 | 1.79E+00 |
| ZCCHC17 | 9.08E-04 | 1.40E+00 |
| ZNF593 | 1.02E-03 | -1.46E+00 |
| HIVEP2 | 1.11E-03 | 1.25E+00 |
| KCNA3 | 1.13E-03 | 1.32E+00 |
| MID1 | 1.29E-03 | -1.65E+00 |
| VPS13A | 1.33E-03 | 1.29E+00 |
| FAM83H | 1.33E-03 | -1.31E+00 |
| SNORD62A | 1.44E-03 | 5.35E+00 |
| SNORD62B | 1.44E-03 | 5.35E+00 |
| LXN | 1.47E-03 | 1.54E+00 |
| ZNF227 | 1.48E-03 | 1.72E+00 |
| PREPL | 1.51E-03 | 1.43E+00 |
| TIGIT | 1.56E-03 | 1.37E+00 |
| SNORD2 | 1.59E-03 | 1.67E+00 |
| CD3E | 1.61E-03 | 1.33E+00 |
| DAP3 | 1.76E-03 | 1.29E+00 |
| AGAP6 | 1.80E-03 | 1.26E+00 |
| IL7R | 1.88E-03 | 1.32E+00 |
| TERF2 | 1.96E-03 | 1.25E+00 |
| SNORD94 | 2.05E-03 | 1.48E+00 |
| AOC2 | 2.13E-03 | -1.67E+00 |
| SNORD14E | 2.18E-03 | 1.49E+00 |
| GLS | 2.23E-03 | 1.21E+00 |
| OPRPN | 2.27E-03 | 8.63E+00 |
| DUOXA1 | 2.40E-03 | -1.30E+00 |
| SLC39A8 | 2.66E-03 | 1.76E+00 |
| SNORD83B | 2.69E-03 | 1.53E+00 |
| LINC00441 | 2.71E-03 | -1.27E+00 |
| SNORA6 | 2.72E-03 | 1.57E+00 |
| CPA6 | 2.76E-03 | 1.20E+00 |
| STARD4 | 2.81E-03 | -1.32E+00 |
| HIGD2A | 2.83E-03 | 1.20E+00 |
| LOC103611081 | 2.85E-03 | 2.91E+00 |
| HMGN2 | 2.89E-03 | -1.26E+00 |
| SNORA54 | 2.91E-03 | 1.75E+00 |
| SNORA5A | 2.93E-03 | 1.46E+00 |
| CCDC148 | 2.94E-03 | 1.49E+00 |
| BHMG1 | 3.16E-03 | -1.33E+00 |
| SNORD91B | 3.16E-03 | 1.54E+00 |
| RGCC | 3.29E-03 | -1.22E+00 |
| SNORD11B | 3.42E-03 | 1.36E+00 |
| SNORD80 | 3.54E-03 | 1.38E+00 |
| SNORD91A | 3.55E-03 | 1.45E+00 |
| VSIG1 | 3.64E-03 | 1.58E+00 |
| SNORD60 | 3.72E-03 | 1.52E+00 |
| ZNF24 | 3.74E-03 | 1.36E+00 |
| SKOR2 | 3.81E-03 | -1.85E+00 |
| ZNF337 | 3.82E-03 | 1.28E+00 |
| RFT1 | 3.86E-03 | -1.26E+00 |
| CD207 | 3.97E-03 | 1.73E+00 |
| AKT3 | 3.97E-03 | 1.22E+00 |
| LOC101929516 | 4.00E-03 | 1.73E+00 |
| GRK4 | 4.07E-03 | -1.30E+00 |
| HIST1H4K | 4.07E-03 | 1.24E+00 |
| SNORD84 | 4.14E-03 | 1.53E+00 |
| ANG | 4.30E-03 | -1.29E+00 |
| BCL11B | 4.39E-03 | 1.31E+00 |
| LEF1 | 4.57E-03 | 1.48E+00 |
| TSSK3 | 4.58E-03 | -1.44E+00 |
| ADSL | 4.59E-03 | 1.51E+00 |
| CCR8 | 4.67E-03 | 3.18E+00 |
| GALR2 | 4.74E-03 | 1.26E+00 |
| SNORA65 | 4.77E-03 | 1.83E+00 |
| SNORD14B | 4.84E-03 | 1.31E+00 |
| BRCA1 | 4.92E-03 | -1.26E+00 |
| C11orf96 | 4.97E-03 | -1.48E+00 |
| HIST1H1D | 5.00E-03 | 1.26E+00 |
| SNORA46 | 5.05E-03 | 3.14E+00 |
| SNORD14C | 5.32E-03 | 1.40E+00 |
| SNHG4 | 5.34E-03 | -1.44E+00 |
| GBX1 | 5.36E-03 | 1.22E+00 |
| LOC101929574 | 5.46E-03 | 1.31E+00 |
| SNORA17B | 5.46E-03 | 1.59E+00 |
| SNURF | 5.49E-03 | 1.21E+00 |
| GJD3 | 5.50E-03 | 1.37E+00 |
| CDR2 | 5.52E-03 | 1.48E+00 |
| ZNF792 | 5.54E-03 | 1.27E+00 |
| LINC02141 | 5.63E-03 | 1.52E+00 |
| TSSC2 | 5.71E-03 | -1.27E+00 |
| CCNC | 5.74E-03 | -1.22E+00 |
| OR2M2 | 5.92E-03 | -3.60E+00 |
| THEMIS | 5.93E-03 | 1.45E+00 |
| ZNF283 | 5.95E-03 | -1.21E+00 |
| HP | 5.97E-03 | -1.87E+00 |
| SNORD5 | 6.12E-03 | 1.45E+00 |
| SNORD9 | 6.35E-03 | 1.76E+00 |
| NOCT | 6.38E-03 | 1.22E+00 |
| CD96 | 6.62E-03 | 1.32E+00 |
| LOC100507634 | 6.68E-03 | -1.34E+00 |
| ZNF3 | 6.68E-03 | 1.36E+00 |
| SNORD35B | 6.83E-03 | 1.24E+00 |
| PGPEP1 | 6.91E-03 | 1.26E+00 |
| NTN1 | 7.04E-03 | -1.27E+00 |
| ZNF396 | 7.12E-03 | -1.79E+00 |
| SCARNA6 | 7.15E-03 | 1.52E+00 |
| TCF7 | 7.39E-03 | 1.24E+00 |
| LOC101928150 | 7.50E-03 | 1.30E+00 |
| DAO | 7.51E-03 | -1.23E+00 |
| ZNF8 | 7.56E-03 | 1.26E+00 |
| AKR7A2 | 7.61E-03 | -1.23E+00 |
| SLC39A1 | 7.64E-03 | -1.20E+00 |
| TRAT1 | 7.64E-03 | 1.39E+00 |
| LOC100507387 | 7.70E-03 | 1.28E+00 |
| SNORD57 | 7.77E-03 | 1.37E+00 |
| SCARNA17 | 8.06E-03 | 1.23E+00 |
| ICOS | 8.26E-03 | 2.12E+00 |
| HNRNPA1P10 | 8.29E-03 | 1.24E+00 |
| SIGLECL1 | 8.39E-03 | 6.88E+00 |
| SNORD24 | 8.52E-03 | 1.38E+00 |
| PIK3IP1-AS1 | 8.54E-03 | 3.86E+00 |
| CASP12 | 8.80E-03 | -2.46E+00 |
| C1orf229 | 8.85E-03 | -1.33E+00 |
| SLC4A7 | 8.89E-03 | 1.26E+00 |
| ZBTB45 | 8.93E-03 | -1.21E+00 |
| SPAG4 | 8.94E-03 | -1.49E+00 |
| SNORD117 | 9.04E-03 | 1.61E+00 |
| CYYR1-AS1 | 9.11E-03 | 4.80E+00 |
| FAM210A | 9.15E-03 | -1.28E+00 |
| MIR193A | 9.17E-03 | -1.31E+00 |
| C1GALT1C1 | 9.18E-03 | -1.51E+00 |
| GPR35 | 9.22E-03 | -1.29E+00 |
| TMEM208 | 9.43E-03 | 1.26E+00 |
| KLK1 | 9.53E-03 | -1.23E+00 |
| CD200R1 | 9.55E-03 | 1.22E+00 |
| SLC33A1 | 9.66E-03 | 1.35E+00 |
| SHH | 9.67E-03 | -1.29E+00 |
| SNORA70 | 9.69E-03 | 1.45E+00 |
| TEX9 | 9.89E-03 | -1.31E+00 |
| MCEMP1 | 9.92E-03 | -2.33E+00 |
| EID3 | 9.95E-03 | -1.51E+00 |
| CEP97 | 9.97E-03 | -1.28E+00 |
| ASZ1 | 9.97E-03 | 1.57E+00 |
| HS3ST4 | 9.98E-03 | -1.33E+00 |

**Supplemental Table 2.** 100 Differentially expressed genes (p-value <0.01; |fold change| ≥ 1.2) in control-smokers (C-SM) vs control-never smokers (C-NSM).

| Gene ID | P-value (C-SM vs C-NSM) | Fold change (C-SM vs C-NSM) |
| --- | --- | --- |
| LRRD1 | 9.59E-03 | 5.12E+01 |
| C10orf120 | 2.66E-04 | 2.91E+01 |
| TTTY19 | 7.43E-03 | 2.51E+01 |
| ZNF716 | 6.74E-03 | 1.98E+01 |
| GPR15 | 2.35E-24 | 1.65E+01 |
| SNORD115-2 | 9.19E-03 | 1.31E+01 |
| SNORD116-12 | 8.23E-03 | 1.19E+01 |
| LOC100507071 | 8.11E-03 | 9.31E+00 |
| SPINK6 | 9.86E-03 | 8.40E+00 |
| ARL6 | 8.17E-03 | 5.28E+00 |
| LOC105376575 | 1.55E-04 | 5.14E+00 |
| SNRPD1 | 1.58E-03 | 5.09E+00 |
| XIRP2-AS1 | 7.51E-04 | 5.06E+00 |
| SNORD1B | 4.99E-04 | 5.06E+00 |
| SDC4 | 4.66E-04 | 4.51E+00 |
| ICOS | 4.44E-03 | 4.37E+00 |
| LOC105376736 | 1.16E-03 | 4.22E+00 |
| CNTN6 | 8.44E-03 | 4.07E+00 |
| LINC00347 | 4.54E-03 | 3.57E+00 |
| LRRN3 | 3.06E-06 | 3.19E+00 |
| LINC00354 | 1.25E-03 | 2.99E+00 |
| RNASE2 | 3.24E-03 | 2.87E+00 |
| CACNG3 | 8.43E-03 | 2.79E+00 |
| ECRP | 1.40E-05 | 2.75E+00 |
| ZNF845 | 2.60E-03 | 2.45E+00 |
| FMN1 | 5.25E-03 | 2.43E+00 |
| CCDC112 | 2.66E-03 | 2.23E+00 |
| EIF2B3 | 2.40E-03 | 2.14E+00 |
| LY6E | 4.06E-03 | 2.12E+00 |
| GTF2H3 | 7.38E-03 | 2.09E+00 |
| LPAR6 | 6.14E-03 | 2.04E+00 |
| NSMCE3 | 4.46E-03 | 2.03E+00 |
| TM2D2 | 6.17E-03 | 1.91E+00 |
| GCNT4 | 9.14E-03 | 1.91E+00 |
| APOL1 | 7.80E-03 | 1.66E+00 |
| VRK1 | 8.98E-03 | 1.64E+00 |
| GUF1 | 3.12E-04 | 1.63E+00 |
| INTS14 | 5.29E-03 | 1.62E+00 |
| RNU11 | 4.08E-03 | 1.62E+00 |
| SLF2 | 6.56E-03 | 1.61E+00 |
| RPL13 | 7.10E-03 | 1.52E+00 |
| VPS13A | 2.94E-03 | 1.51E+00 |
| HIST1H4C | 5.48E-03 | 1.50E+00 |
| DAP3 | 6.48E-04 | 1.49E+00 |
| OARD1 | 3.07E-03 | 1.49E+00 |
| TRAPPC2L | 5.39E-03 | 1.48E+00 |
| IFI27L2 | 6.01E-03 | 1.48E+00 |
| RPL29 | 6.84E-03 | 1.48E+00 |
| IL2RA | 6.50E-04 | 1.45E+00 |
| DCLRE1C | 5.25E-03 | 1.45E+00 |
| CLDND1 | 4.12E-05 | 1.43E+00 |
| HIST1H1D | 6.38E-03 | 1.43E+00 |
| SNORD116-22 | 2.75E-03 | 1.38E+00 |
| HIST1H4B | 6.97E-03 | 1.38E+00 |
| TNFSF8 | 3.82E-03 | 1.37E+00 |
| SNORD1C | 7.62E-03 | 1.37E+00 |
| SNORA61 | 4.22E-03 | 1.35E+00 |
| SCARNA6 | 8.40E-03 | 1.35E+00 |
| SNORD76 | 5.98E-03 | 1.34E+00 |
| CBR3-AS1 | 2.69E-03 | 1.33E+00 |
| RAB44 | 1.30E-03 | 1.33E+00 |
| SNORD116-15 | 2.75E-03 | 1.32E+00 |
| ZNF75A | 9.45E-03 | 1.31E+00 |
| SNORA8 | 9.40E-03 | 1.30E+00 |
| SNORD3C | 2.15E-03 | 1.28E+00 |
| SNORD99 | 4.15E-03 | 1.28E+00 |
| SNORD116-3 | 3.15E-03 | 1.27E+00 |
| SNORD116-9 | 3.15E-03 | 1.27E+00 |
| SNORA54 | 8.96E-03 | 1.27E+00 |
| PAICS | 5.27E-03 | 1.26E+00 |
| HIST1H1C | 2.81E-03 | 1.24E+00 |
| SNORD116-14 | 4.75E-03 | 1.23E+00 |
| SNORD110 | 8.68E-03 | 1.23E+00 |
| SNORD29 | 4.62E-03 | 1.22E+00 |
| SNORA16A | 7.80E-03 | 1.22E+00 |
| RUNDC1 | 9.11E-03 | -1.21E+00 |
| LOC254896 | 2.10E-03 | -1.23E+00 |
| APAF1 | 7.47E-03 | -1.23E+00 |
| TOR4A | 8.37E-03 | -1.23E+00 |
| SPAG9 | 9.07E-03 | -1.23E+00 |
| UBN1 | 8.30E-03 | -1.25E+00 |
| PRKAR1A | 1.83E-03 | -1.26E+00 |
| MAN1A1 | 9.40E-03 | -1.28E+00 |
| LRRC4 | 8.38E-03 | -1.28E+00 |
| PAK1 | 6.28E-03 | -1.29E+00 |
| TNFRSF10D | 4.45E-03 | -1.29E+00 |
| CD46 | 5.90E-03 | -1.30E+00 |
| F11R | 5.52E-03 | -1.30E+00 |
| RAB11FIP1 | 3.05E-03 | -1.30E+00 |
| LAMTOR4 | 8.88E-03 | -1.31E+00 |
| LRRK2 | 8.47E-03 | -1.31E+00 |
| SEC14L1P1 | 7.48E-03 | -1.32E+00 |
| EGLN1 | 8.87E-03 | -1.33E+00 |
| MSL1 | 5.13E-03 | -1.33E+00 |
| DNAJC3 | 2.44E-03 | -1.33E+00 |
| MME | 3.59E-03 | -1.39E+00 |
| ADGRE3 | 8.14E-03 | -1.44E+00 |
| SH2D1B | 4.26E-03 | -1.82E+00 |
| KIR3DL1 | 6.35E-03 | -2.04E+00 |
| KIR2DL4 | 1.81E-03 | -2.31E+00 |

**Supplemental Table 3.** Top functional pathways for regulated genes associated with IS-SM when compared to IS-NSM.

| KEGG | Canonical Name | Entrez Gene neighbors | Selected Base Neighbors | Selected Enrichment |
| --- | --- | --- | --- | --- |
| 4660 | T cell receptor signaling pathway | 108 | 5 | 4.91E-06 |
| 4062 | Chemokine signaling pathway | 189 | 5 | 7.25E-05 |
| 512 | Mucin type O-Glycan biosynthesis | 30 | 2 | 0.002089184 |
| 472 | D-Arginine and D-ornithine metabolism | 1 | 1 | 0.002245472 |
| 250 | Alanine, aspartate and glutamate metabolism | 32 | 2 | 0.002375163 |
| 260 | Glycine, serine and threonine metabolism | 32 | 2 | 0.002375163 |
| 4530 | Tight junction | 132 | 3 | 0.003358303 |
| 330 | Arginine and proline metabolism | 55 | 2 | 0.006875435 |
| 471 | D-Glutamine and D-glutamate metabolism | 4 | 1 | 0.008951902 |
| 4920 | Adipocytokine signaling pathway | 68 | 2 | 0.010348559 |
| 4520 | Adherens junction | 73 | 2 | 0.01185182 |
| 1100 | Metabolic pathways | 1131 | 7 | 0.014258622 |
| 4060 | Cytokine-cytokine receptor interaction | 275 | 3 | 0.024394945 |
| 604 | Glycosphingolipid biosynthesis - ganglio series | 15 | 1 | 0.033161567 |
| 4120 | Ubiquitin mediated proteolysis | 135 | 2 | 0.037285054 |
| 4310 | Wnt signaling pathway | 150 | 2 | 0.045081308 |
| 4630 | Jak-STAT signaling pathway | 155 | 2 | 0.047802593 |

**Supplemental Table 4.** Top functional pathways for regulated genes associated with C-SM compared to C-NSM.

| KEGG | Canonical Name | Entrez Gene neighbors | Selected Base Neighbors | Selected Enrichment |
| --- | --- | --- | --- | --- |
| 4650 | Natural killer cell mediated cytotoxicity | 139 | 5 | 1.74E-06 |
| 4210 | Apoptosis | 87 | 3 | 2.65E-04 |
| 4514 | Cell adhesion molecules (CAMs) | 133 | 3 | 9.16E-04 |
| 4612 | Antigen processing and presentation | 76 | 2 | 0.00526037 |
| 4060 | Cytokine-cytokine receptor interaction | 275 | 3 | 0.00709314 |
| 4660 | T cell receptor signaling pathway | 108 | 2 | 0.01035788 |
| 4144 | Endocytosis | 201 | 2 | 0.03312005 |

**Supplemental Figure 1.** Top functional pathways for regulated genes in blood associated with C-SM compared to C-NSM.

Abbreviations: PRKAR1A, Protein Kinase CAMP-Dependent Type I Regulatory Subunit Alpha; APAF1, Apoptotic Peptidase Activating Factor 1; TNFRSF10D, TNF Receptor Superfamily Member 10d; TNFSF8, TNF Superfamily Member 8; KIR2DL4, Killer Cell Immunoglobulin Like Receptor Two Ig Domains And Long Cytoplasmic Tail 4; KIR3DL1, Killer Cell Immunoglobulin Like Receptor; SH2D1B, SH2 Domain Containing 1B; IL2RA, Interleukin 2 Receptor Subunit Alpha; SDC4, Syndecan 4; F11R, F11 Receptor; ICOS, Inducible T Cell Costimulator; MME, Membrane Metalloendopeptidase; PAK1, P21 (RAC1) Activated Kinase 1; LRRK2, Leucine Rich Repeat Kinase 2; SPAG9, Sperm Associated Antigen 9; RAB11FIP1, RAB11 Family Interacting Protein 1.
